# From 3DGS scenes to plant traits: a scalable extraction and segmentation framework for muskmelon phenotyping

**DOI:** 10.3389/fpls.2026.1783465

**Published:** 2026-04-16

**Authors:** Jing-Heng Lin, Ta-Te Lin

**Affiliations:** Department of Biomechatronics Engineering, National Taiwan University, Taipei, Taiwan

**Keywords:** 3D gaussian splatting, 3D reconstruction, instance segmentation, plant phenotyping, trait extraction

## Abstract

Automated quantification of plant-level development from multi-plant greenhouse scenes requires separating individual plants from shared scene-level reconstructions and quantifying organ-level development, a challenge that single-plant acquisition workflows do not directly address. This study presents an end-to-end phenotyping pipeline built on 3D Gaussian Splatting (3DGS) and a post-reconstruction extraction framework, LCR-GS, designed to isolate plant instances from full greenhouse scenes without scene-specific model retraining. LCR-GS integrates zero-shot 2D cues with multi-view lifting, geometric clustering, and chromatic refinement to convert large scene-level reconstructions (~2M Gaussians) into compact per-plant subsets (~16K Gaussians). Experiments on greenhouse-grown muskmelon at the early vegetative stage demonstrate high plant-extraction precision (0.933) and strong organ-level instance segmentation (mean AP50 = 0.924). Plant height and leaf count are validated against manual measurements (height R² = 0.98, RMSE = 1.88 cm; leaf count R² = 0.86), whereas additional morphological traits, including leaf area, leaf area index, mean internode length, and stem node count, are reported as pipeline-derived descriptors for within-cohort comparison. By decoupling semantic inference from reconstruction, the pipeline reduces scene-scale data by over 99% and provides a practical route to derive compact per-plant 3D representations from multi-plant greenhouse imagery for downstream organ-level analysis.

## 1 Introduction

Phenotypic characterization of high-value horticultural crops is essential for optimizing yield, quality, and management decisions in modern agriculture. Although traditional manual measurements remain accurate, they are labor-intensive and inherently limited in throughput, restricting their use in large-scale breeding or production programs (Araus et al., 2018). Image-based phenotyping systems have therefore been widely adopted as an effective alternative, enabling rapid, non-destructive trait quantification across development stages (Li et al., 2014; Wang et al., 2025). However, 2D imagery cannot fully recover the volumetric structure needed to resolve overlapping foliage, occluded organs, and plant-level geometry in shared multi-plant scenes. In greenhouse-grown muskmelon, neighboring leaves, vines, and support structures can overlap in image space even under controlled acquisition, making it difficult to separate individual plants and recover organ-level geometry from 2D views alone. Many important traits, including canopy architecture, biomass distribution, and organ-level geometry, therefore require three-dimensional reconstruction for accurate assessment (Akhtar et al., 2024). Recent progress in mobile platforms, including unmanned ground vehicles (UGVs) and unmanned aerial systems (UAS), has expanded access to large-scale 3D phenotyping (Feng et al., 2021; Rui et al., 2024). Achieving useful plant-level analysis from such acquisitions, however, still depends on 3D reconstruction and analysis pipelines that can process shared multi-plant scenes while preserving individual-plant resolution.

Extracting phenotypic traits from 3D plant reconstructions requires robust segmentation and geometric analysis. Recent progress in point-based deep learning has substantially advanced this capability. Foundational feature extractors such as PointNet/PointNet++ (Qi et al., 2017a, 2017) and DGCNN (Wang et al., 2019) established methods for permutation-invariant feature learning, while Transformer-based backbones (Wu et al., 2024; Zhao et al., 2021) further improved the modeling of complex geometric relationships. Complementing these feature extractors, general-purpose segmentation architectures, such as PointGroup’s offset clustering (Jiang et al., 2020), provided reliable mechanisms for separating discrete plant organs. Together, these technologies form the basis for many plant-specific 3D phenotyping applications.

Building on these general-purpose architectures, plant-specific 3D analysis methods have evolved into three major functional categories. First, semantic segmentation classifies point clouds into organ types for species-level architectural analysis (Li et al., 2022a; Shi et al., 2019). Second, instance segmentation, which separates individual organs, is critical for tasks such as leaf counting, organ tracking, and per-organ trait measurement. This capability has been demonstrated across a wide range of crops, including maize (Li et al., 2022b), rapeseed (Du et al., 2023), and wheat (Ghahremani et al., 2021). Alongside these, other specialized techniques such as graph-based topological reasoning (Mirande et al., 2022) and multi-view fusion (Shi et al., 2019) have been developed to address occlusion and structural ambiguity in complex plant scenes. Third, geometric processing complements segmentation by directly extracting morphological traits: skeleton-based methods quantify branching topology and internode spacing (Cao et al., 2010), while mesh-based approaches estimate leaf area and surface curvature (Boukhana et al., 2022). Despite their diversity, these methods all rely on a critical but often implicit assumption: the availability of clean, pre-isolated, single-plant point clouds. While this condition is easily met in controlled environments, it becomes a major bottleneck in structured multi-plant greenhouse scenes, where neighboring plants and surrounding greenhouse structures are reconstructed together. This extraction step represents an intermediate bottleneck in phenotyping pipelines, where scene-level reconstructions must be decomposed into plant-level units before organ-level segmentation and trait computation can be applied.

The practical effectiveness of 3D segmentation, therefore, depends critically on robust and accessible reconstruction workflows. Although LiDAR remains the precision benchmark, its cost, operational complexity, and limited scalability make it unsuitable for large multi-plot phenotyping programs. In contrast, RGB-based reconstruction, typically performed using Structure from Motion (SfM) tools such as COLMAP (Schönberger and Frahm, 2016), offers a cost-effective and flexible alternative for deployment on UGVs and UAVs. Recent advances in neural rendering have further improved reconstruction quality: Neural Radiance Fields (NeRF) (Mildenhall et al., 2021) provides photorealistic novel view synthesis and improved geometric consistency in agricultural applications (Arshad et al., 2024; Choi et al., 2024). However, NeRF’s continuous volumetric representation lacks explicit geometry, creating a fundamental mismatch with downstream pipelines that require measurable, discrete structures for segmentation and trait extraction. 3D Gaussian Splatting (3DGS) addresses this gap by representing scenes as spatially explicit Gaussian primitives (Kerbl et al., 2023), offering NeRF-level fidelity while maintaining compatibility with point-based analysis. Recent plant-focused adaptations (Jiang et al., 2025; Shen et al., 2025) further highlight 3DGS’s suitability for phenotyping applications.

In multi-plant phenotyping applications of 3D Gaussian Splatting (3DGS), two practical challenges arise. First, shared scene reconstructions contain neighboring plants and surrounding structures, making it difficult for downstream workflows, which typically assume clean, single-plant geometry, to operate reliably (Harandi et al., 2023). Second, and more specific to 3DGS, contemporary scene reconstructions consist of millions of Gaussian primitives (Fang and Wang, 2024; Li et al., 2025b). This high density, combined with noise introduced by sparse or uneven view coverage, makes direct point-based feature extraction computationally expensive and reduces geometric consistency. Recent work has attempted to address these issues by injecting 2D semantic cues during 3DGS optimization to obtain promptable or open-world segmentation. Gaussian Grouping lifts multi-view 2D features into the Gaussian space and learns instance groupings during reconstruction (Ye et al., 2024), whereas SAGA (Segment Any 3D Gaussians) distills 2D foundation models into Gaussian features to enable prompTable 3D segmentation at inference (Cen et al., 2025). While effective for general 3D scene editing, these training-integrated approaches couple semantic learning with reconstruction, limiting flexibility and increasing computational cost. These limitations motivate a post-reconstruction strategy that decouples reconstruction from semantic assignment so that a reconstructed scene can be reused for plant extraction without modifying the 3DGS model itself.

The validated setting in this study occupies a middle regime between two relevant reference points. One recent object-centric 3DGS phenotyping workflow for strawberry isolates individual plants before reconstruction to obtain clean single-plant models (Li et al., 2026). At the other end, recent surveys of plant 3D reconstruction and 3DGS-based phenotyping note that dense, highly entangled canopies remain challenging for current pipelines (Li et al., 2025a). Our focus lies between these extremes: multiple neighboring greenhouse plants share one scene reconstruction, canopy entanglement remains limited, and downstream analysis still requires that the shared reconstruction be decomposed into individual plant units. This structured shared-scene extraction problem is narrower than dense-canopy phenotyping, but it is not addressed by object-centric single-plant workflows and remains underexplored in current 3DGS phenotyping studies.

To address this shared-scene decomposition problem, we propose LCR-GS (Lift, Cluster, Refine for Gaussian Splat), a post-reconstruction framework that operates on fixed 3DGS reconstructions and introduces semantics via zero-shot 2D foundation models. The pipeline employs YOLO-World detections and SAM (Segment Anything Model) with minimal operator seeding on a small subset of views to initialize a reusable pool of multi-view cues (Cheng et al., 2024; Kirillov et al., 2023). The seeded cues are then lifted to 3D Gaussians with multi-view projection and consistency filtering, followed by geometric clustering that consolidates spatially coherent groups and isolates target plant instances while suppressing background structures. The resulting per-plant representations are then used for point-based organ-level instance segmentation and phenotypic trait estimation.

In this study, our objective is to establish a bounded workflow that bridges scene-level 3D reconstruction and plant-level trait quantification for structured multi-plant greenhouse phenotyping. We make three main contributions toward this goal. First, we develop an end-to-end phenotyping pipeline based on three-dimensional Gaussian Splatting that reconstructs full greenhouse scenes while preserving per-plant spatial resolution for downstream organ-level analysis. Second, we introduce LCR-GS, a post-reconstruction extraction framework that decouples semantic inference from 3DGS optimization. LCR-GS requires only minimal operator seeding at initialization and then automatically performs multi-view lifting, geometric clustering, and refinement to convert structured multi-plant greenhouse scenes into clean, instance-level plant representations. This design enables reconstruction reuse, reduces annotation effort, and transforms scene-scale models into analysis-ready point sets. Third, we show that these extracted plant instances support point-based organ segmentation and bounded quantitative trait estimation for greenhouse-grown muskmelon. Validation on early-vegetative greenhouse muskmelon shows that the proposed framework provides a practical route from 3DGS scene reconstruction to organ-level trait quantification within this study setting.

## 2 Materials and methods

### 2.1 Pipeline overview

**Figure 1:**
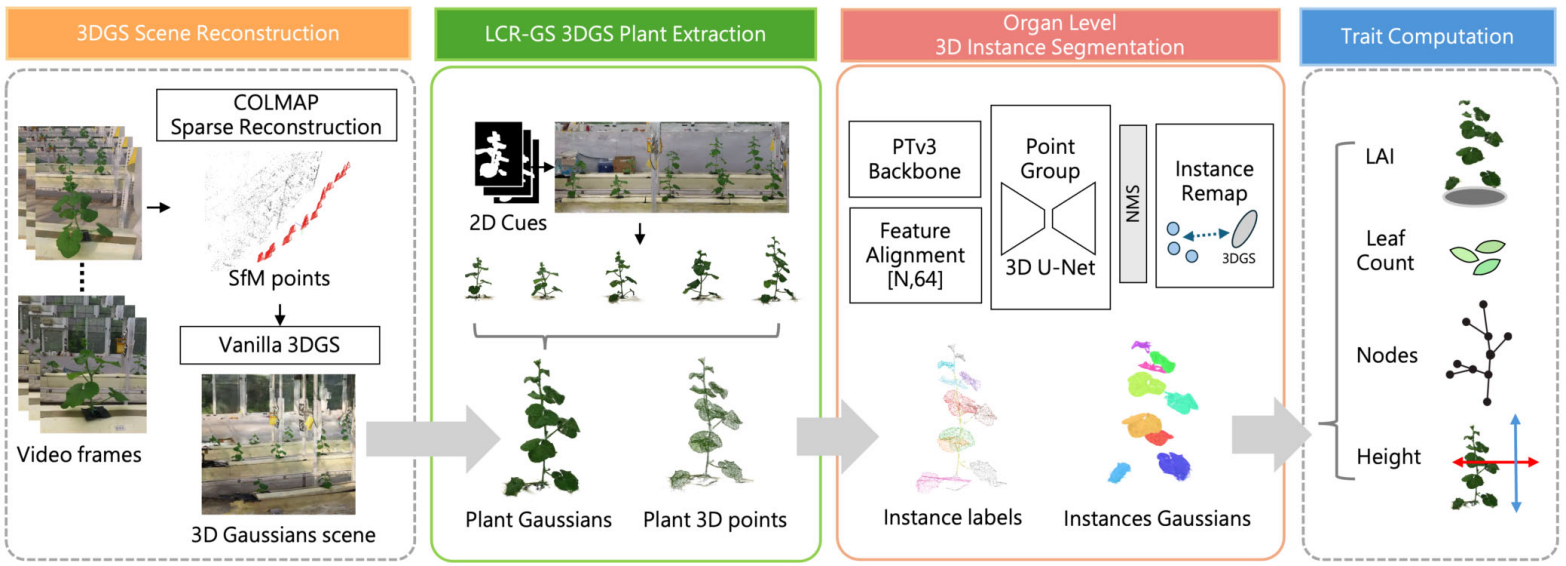
Overview of the end-to-end phenotyping pipeline based on 3D Gaussian Splatting (3DGS).

**Figure 1** provides an overview of the proposed end-to-end pipeline for structured multi-plant greenhouse phenotyping, which consists of four stages: (1) multi-view RGB acquisition and Structure-from-motion, followed by 3D Gaussian Splatting to obtain a fixed scene reconstruction (Section 2.2); (2) the core LCR-GS post reconstruction extraction that lifts two dimensional cues to three-dimensional Gaussians, clusters, and refines the result to isolate individual plants (Section 2.3); (3) organ level instance segmentation on exported point clouds, with segmentation results remapped to the 3DGS domain (Section 2.4); and (4) trait computation on the segmented instances (Section 2.5). The following subsections first describe data acquisition and reconstruction, then present the LCR-GS extraction procedure, and finally detail the segmentation and trait computation modules.

### 2.2 Data acquisition and 3DGS scene reconstruction

#### 2.2.1 Data acquisition protocol and dataset

Greenhouse data were collected at the National Taiwan University Experimental Farm in a muskmelon (Cucumis melo L.) greenhouse in August 2024. Video was recorded with an Insta360 One RS equipped with the Ultrawide 80 lens at 3840×2160 and 30 fps. Electronic stabilization was disabled to preserve geometric consistency. For each crop row, two passes were performed: an outbound pass at approximately 0.5 m camera height and a return pass at approximately 1.5 m, ensuring dense multi-view coverage of the canopy. Plants were arranged in a fixed grid layout with five plants per row, a within-row spacing of 72 cm, and a between-row spacing of approximately 1.0-1.2 m. The complete dataset is organized into two distinct subsets, as summarized in **Table 1**.

**Table 1:** The structure and characteristics of the muskmelon dataset.

Subset name	Scenes	Plants	Biological stage	Annotation type
Phenotyping Subset	6	30	Early vegetative	Plant/background labels (Gaussian-level)
Segmentation Training Subset	30	140	Diverse stages/conditions	3D point-cloud organ labels (Point-level)

As indicated in **Table 1**, the phenotyping subset contains 6 greenhouse scenes (30 plants) captured 14–15 days after transplanting, during the early vegetative stage characterized by rapid leaf expansion and internode elongation. This subset is held out from segmentation network training and is used to validate the complete pipeline from plant extraction through trait estimation. For this subset, each Gaussian in the 3DGS reconstruction was manually labeled as plant or background using the SuperSplat Gaussian Splats Editor, which supports point-wise selection and carving within a 3DGS scene.

The segmentation training subset contains 30 additional scenes (140 plants) collected at the same greenhouse facility using the same overall acquisition protocol and 3DGS reconstruction workflow, spanning diverse growth stages and illumination conditions. The two subsets do not overlap: the phenotyping subset was held out from segmentation-model training and reserved for end-to-end pipeline validation.

#### 2.2.2 Structure-from-Motion and 3DGS scene initialization

To balance pose accuracy with computational efficiency, the recorded videos were temporally subsampled before Structure-from-Motion (SfM) processing. Frame quality was evaluated using image sharpness, measured by the variance of the Laplacian, and a temporal coverage constraint enforced uniform spatial progression along the acquisition path. Within each short window, the sharpest frame was retained, provided that inter-frame motion yielded sufficient parallax. COLMAP was then used to estimate calibrated camera intrinsics and extrinsics and to reconstruct a sparse, geometrically consistent point cloud that served as the initialization scaffold for 3D Gaussian Splatting (3DGS).

Building on this SfM output, the next step was to construct the full Gaussian scene by transforming each sparse point into a Gaussian primitive. Each sparse SfM point was converted into a Gaussian primitive defined by its mean *μ*_i_, initial covariance *Σ*_i_ (which becomes anisotropic during optimization), color *c*_i_, and opacity *α*_i_, forming the parameter set *θ* = {*μi*, *Σi*, *αi*, *ci*}. The 3DGS optimization learned *θ* by minimizing a photometric reconstruction loss with opacity and scale regularization, using differentiable rendering through transmittance-weighted anisotropic Gaussian splats. Because anisotropic kernels naturally align with planar foliage, the representation effectively captures thin leaf structures. Each scene was optimized for 7, 000 iterations using the standard densification schedule, producing a fixed, high-fidelity Gaussian reconstruction used for subsequent plant extraction and organ-level segmentation.

### 2.3 Plant gaussians extraction (LCR-GS)

#### 2.3.1 Plant extraction algorithm overview

**Figure 2:**
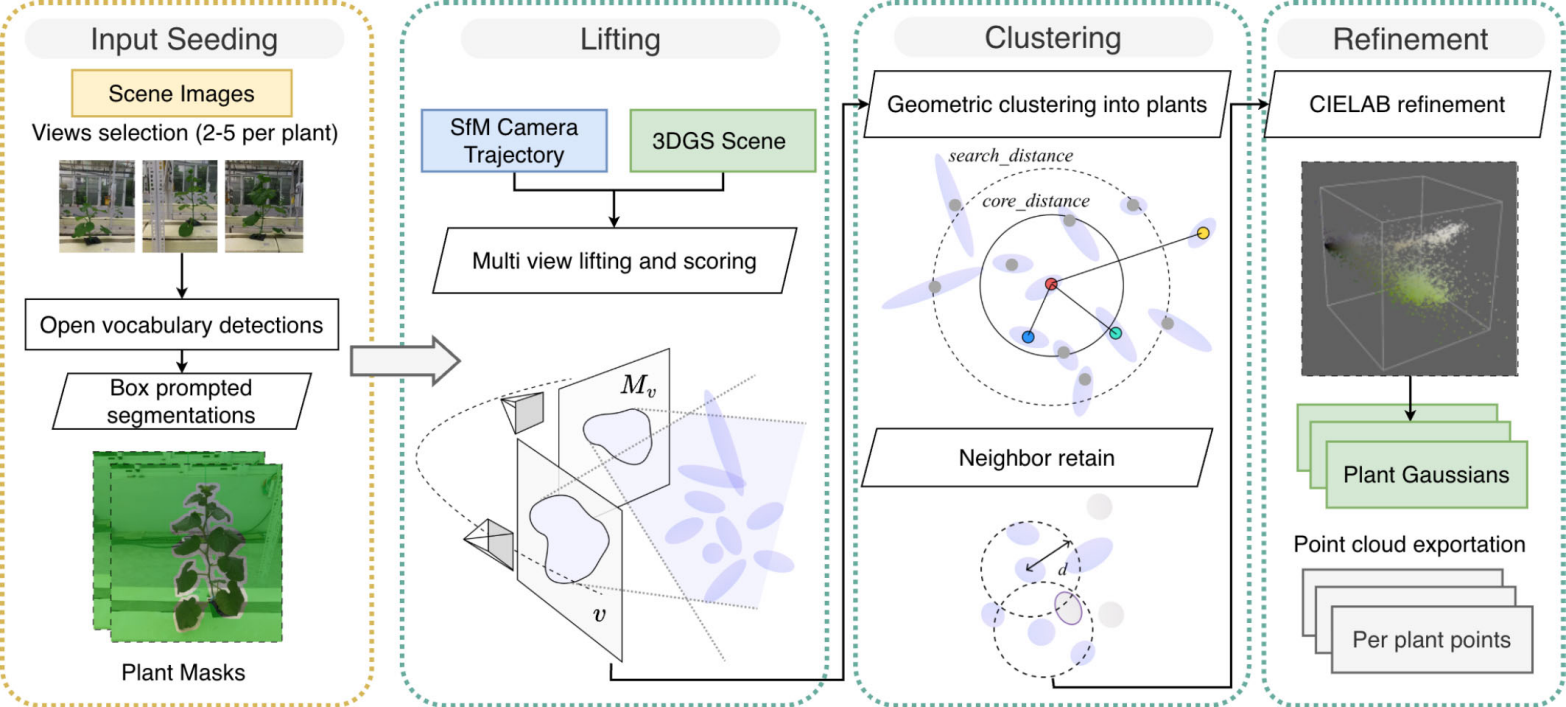
The flowchart illustrates the complete data flow from seed preparation on the left through the three-stage core algorithm on the right (lifting, clustering, refinement), ultimately exporting a 3D plant model.

**Figure 2** illustrates the proposed LCR-GS approach for extracting individual plants from full-scene 3DGS reconstructions. The algorithm consists of a lightweight seeding stage followed by three automated stages - Lift, Cluster, and Refine - which progressively transform 2D semantic cues into a clean, plant-level 3D subset. This design decouples semantic inference from 3DGS optimization, enabling the reuse of fixed Gaussian reconstructions without retraining.

The LCR-GS process begins with a lightweight seeding step, where a small number of manually selected views provide initial 2D semantic masks. These cues are projected into the 3DGS domain to identify Gaussians with multi-view support (Lift). Spatially coherent plant structures are then grouped through geometric clustering and thin-structure restoration (Cluster). A final refinement step uses chromatic filtering in CIELAB color space to remove background remnants and produce clean “Plant Gaussians, “ followed by export to both Gaussian and point-cloud formats (Refine).

#### 2.3.2 Input seeding for LCR-GS initialization

Extraction begins with a one-time, minimal seeding step that initializes the 2D cue pool for each scene. An operator selects one to five representative views per scene that clearly capture the target plant. Open-vocabulary object detections are then applied to these views using YOLO-World, and the detected bounding boxes are passed to SAM to generate box-prompted binary masks. Each resulting cue is stored together with its associated metadata - {image_id, view_index, mask_id, bbox} - where image_id identifies the source frame, view_index its position in the calibrated camera list, mask_id distinguishes multiple masks within the same view, and bbox is the bounding-box prompt passed to SAM. This metadata is cached for reuse throughout the extraction process. These cues provide the only manual input required by LCR-GS: they enable multi-view semantic lifting while avoiding any scene-specific retraining or additional labeling beyond the initial selection.

#### 2.3.3 Lifting 2D cues into the 3DGS domain

This step lifts sparse 2D segmentations into the 3D domain by evaluating how consistently each Gaussian is supported across all selected viewpoints. Because each 2D mask provides only a partial and view-dependent observation of plant structure, multi-view lifting aggregates evidence across viewpoints to establish reliable semantic support. The lifting stage computes, for each Gaussian, how much of its projected footprint is covered by the 2D masks and how much confidence each view contributes.

In 3DGS reconstruction, each Gaussian is associated with a center $\mu_{g}$, covariance ${\text{Σ}}_{g}$, color $c_{g}$, and opacity $\alpha_{g}$. The goal of the lifting stage is to aggregate 2D semantic cues from multiple views and assign a multi-view support score to each Gaussian, indicating how strongly it is supported as belonging to the target plant.

Each Gaussian is projected into all seeded views using the calibrated intrinsics and extrinsics. For the Gaussian g in view v, projection of $\left(\mu_{g},{\text{Σ}}_{g}\right)$ yields an ellipse $E_{g,v}$ with footprint $\text{Ω}(E_{g,v})$. A separable kernel $\kappa_{g,v}$ is normalized to sum to one over the footprint $\left(E_{g,v}\right)$. With a binary cue $M_{v}\in \{0,1\}$, the masked footprint fraction and its opacity weighted score are defined as (Equation 1):

(1) $$\begin{array}{c} {\tilde{s}}_{g,v}=\sum_{u\in \text{Ω}(E_{g,v})}\kappa_{g,v}(u)M_{v}(u),s_{g,v}=\alpha_{g}{\tilde{s}}_{g,v}\;. \end{array}$$

Each view contributes according to a geometry- and quality-dependent weight (Equation 2):

(2) $$w_{v}={\bar{\alpha }}_{g,v}x\text{max}(\text{cos}\theta_{g,v},0)\rho_{v},$$

where ${\bar{\alpha }}_{g,v}$ denotes the mean opacity over $\text{Ω}(E_{g,v})$ as a visibility proxy, $\theta_{g,v}$ is the angle between the viewing ray in view v and a principal axis derived from ${\text{Σ}}_{g}$, and $\rho_{v}$ ∈ [0, 1] is the sharpness score of frame v, computed as the variance of the Laplacian and robustly normalized within each scene by clipping to the 5th-95th percentile range and rescaling to [0, 1].

The multi-view support score is obtained by aggregating the per-view contributions:

Foreground Gaussians are selected by applying a scene-adaptive threshold. Let τ=quantile(S,q) for a chosen percentile q. The foreground set is (Equations 3, 4):

(3) $$\begin{array}{ccc} S(g)=\frac{\sum_{v}w_{v}s_{g,v}}{\sum_{v}w_{v}} & . & \; \end{array}$$

(4) F={g∣S(g)≥τ}.

This foreground set is passed to geometric clustering stage for structural consolidation.

#### 2.3.4 Geometric clustering and retention filtering

Because the lifted foreground set may still contain background fragments or multi-plant overlap, geometric clustering is required to consolidate spatially coherent plant structures. After foreground selection, remaining Gaussians are grouped by OPTICS (Ordering Points To Identify the Clustering Structure) in a four-dimensional feature space that combines world-space coordinates with a view-agnostic depth cue (Ankerst et al., 1999). Let $\mu_{g}={\left(x_{g},y_{g},z_{g}\right)}^{⊤}$denote the world coordinates of Gaussian g. To ensure that clustering responds to plant geometry rather than global scene layout, we first normalize all spatial features. To prevent distances in the feature space from being dominated by raw scene extent or outliers, a robust standardization operator is introduced and applied consistently to all scalar quantities (Equation 5):

(5) $$Z(s|S)=\frac{s-\text{median}(S)}{\text{MAD}(S)},$$

where S is the set of values for a specific feature across all Gaussians, and MAD is the median absolute deviation. This operator centers each feature by its median and rescales by its intrinsic variability, producing standardized values that remain comparable across scenes with different scales or sampling densities. Applying Z(·) to each axis produces standardized coordinates used for shape similarity evaluation (Equation 6):

(6) $$x_{g}^{\prime }=Z\;(x_{g}\{x_{j}\}),y_{g}^{\prime }=Z(y_{g}\{y_{j}\}),z_{g}^{\prime }=Z(z_{g}\{z_{j}\}),$$

where {*x_j_*}, {*y_j_*}, {*z_j_*}represent the sets of coordinate values across all filtered Gaussians. To ensure depth is encoded consistently across viewpoints, a view-agnostic depth cue is computed. Let C(g) be the set of camera centers from which the Gaussian  g is visible. The average camera distance ${\bar{r}}_{g}$ is defined as (Equation 7):

(7) $${\bar{r}}_{g}=\frac{1}{|C(g)|}\sum_{c\in C(g)}∥\mu_{g}-c∥,\;$$

where c represents a specific camera center vector. Standardizing ${\bar{r}}_{g}$ produces a depth feature that is directly comparable to the three coordinate features (Equation 8):

(8) $$d_{g}^{\prime }=Z({\bar{r}}_{g}|\{{\bar{r}}_{j}\}).$$

The complete clustering descriptor $\phi_{g}$ is then assembled by concatenating the four standardized components (Equation 9):

(9) $$\phi_{g}=[x_{g}^{\prime },y_{g}^{\prime },z_{g}^{\prime },d_{g}^{\prime }],$$

so that Euclidean distance balances local geometric shape with the depth cue. OPTICS is applied to the set $\left\{\phi_{g}\right\}$ using the Euclidean metric, and clusters are extracted using the steepness-based ξ -method ( ξ = 0.1), which identifies clusters as valleys in the reachability plot.

To reduce over-segmentation, adjacent clusters are merged when their centroid distance falls within a cluster-adaptive threshold defined as the 90th percentile of point-to-centroid distances in the selected cluster, and when their median depth values differ by less than 15% relative to the selected cluster’s median depth. To recover thin foliage elements that may fall below the initial clustering threshold, a nearest-neighbor retain step restores any rejected Gaussian whose nearest kept neighbor lies within a global radius (Equation 10):

(10) $$r_{\text{nr}}=\beta {\tilde{d}}_{1\text{NN}},$$

where ${\tilde{d}}_{1\text{NN}}$ denotes the median nearest-neighbor distance among the retained Gaussians and β is a fixed scale factor (default β=2). This stage is purely geometric, acting as a robustness filter that consolidates a coherent per-plant subset before the refinement step.

#### 2.3.5 Chromatic refinement and instance exportation

The final refinement step converts the clustered candidate subset into a compact, plant-focused representation by suppressing residual background elements using chromatic filtering. Chromatic outliers are attenuated in the CIELAB (CIE L*a*b*) color space, which separates luminance and chromatic components and is widely used for plant-background separation and color-based analysis (Hernández-Hernández et al., 2016; Pape and Klukas, 2014). A full-covariance Gaussian mixture model (GMM) with up to four components is fitted in CIELAB space, with the number of components selected by the Bayesian Information Criterion (BIC). When the candidate point set exceeds 50, 000 points, a stratified subsample is drawn using quantile-cell sampling in CIELAB space to preserve the overall color distribution; smaller sets are used in full. The dominant component is selected by mixture weight, and an ellipsoidal chromaticity window is defined by a single coverage parameter ζ∈(0,1). Gaussians whose Mahalanobis distance to the dominant component falls inside this window are retained (Equation 11):

(11) $$D_{M}^{2}(x)\leq \tau (\zeta ),$$

where τ(ζ) is the ζ-quantile of the in-component Mahalanobis distance distribution. Unless otherwise noted, ζ=0.80 is used. During training, a relaxed coverage ζ=0.90 is used to increase recall for organ-level segmentation; the stricter default value is reinstated when remapping instance-level results back to the 3DGS representation. This refinement procedure is fully automatic and scene adaptive, requiring no manual threshold setting.

The refined plant subset is exported in two complementary forms. First, a dense 3D point cloud is generated by sampling each Gaussian ellipsoid in its principal-axis frame, with the sample count proportional to opacity. Samples are mapped to world coordinates and inherit renderer colors, producing a clean and color-consistent point set for point-based learning (Stuart et al., 2025). Second, the corresponding Gaussian subset is preserved to support rendering and to remap organ-level segmentation results back into the original 3DGS representation for downstream phenotypic analysis.

### 2.4 Organ-Level instance segmentation

#### 2.4.1 Input normalization and organ instance segmentation model

Per-plant point clouds exported from the 3DGS representation are segmented into stems and leaves using a transformer-based point cloud network with a PointGroup-style bottom up head. Because reconstructions derived from structure-from-motion lack a consistent metric scale, all inputs are normalized before learning to ensure uniform spatial density across scenes. Let $x_{i}$ denote a raw point, c the median spatial centroid, and s the median k-nearest-neighbor spacing. Each point is normalized to a target spacing Δ by (Equation 12)

(12) $$x_{i}^{\prime }=c+(x_{i}-c)\cdot \frac{\text{Δ}}{s},$$

which stabilizes voxel resolution, attention coverage, and grouping radii across scenes. This normalization addresses variation in point cloud density across scenes and is distinct from the subsequent metric scale alignment step.

The segmentation backbone follows Point Transformer V3 (PTv3), which preserves permutation invariance while capturing long-range context through attention on serialized local neighborhoods and radius-aware downsampling. The network outputs per-point feature descriptors and semantic logits for stems and leaf classes. Training uses AdamW with mixed precision; the semantic head is trained with a base learning rate of and weight decay under a one-cycle schedule with warm-up, while the backbone is fine-tuned at a reduced learning rate of. The global batch size is 12, and training runs for 800 epochs. This organ-segmentation model is trained once for the target crop setting and subsequently applied to all extracted plant instances without scene-specific retraining or fine-tuning.

Instance grouping follows a PointGroup-style bottom-up procedure. For each semantic class k, the subset $S_{k}=\{i|{\hat{y}}_{i}=k\}$ is partitioned into connected components using a Euclidean radius expressed relative to density, r=γ Δ, where γ is a fixed scale factor. Two points in $S_{k}$ are linked when $∥x_{i}^{\prime }-x_{j}^{\prime }∥\leq r$. A breadth first search aggregates links into spatially contiguous regions representing individual organs. Instance confidence is computed as the average semantic logit across its member points.

#### 2.4.2 Remapping segmentations to the 3DGS domain

Per-point labels from the normalized cloud are transferred back to the original 3DGS reconstruction for visualization and downstream trait computation. Only visible splats are considered, as determined by their opacity parameter ω using $\sigma (\omega )=1/(1+e^{-\omega })$ with a small threshold τ. For each visible splat, the nearest labeled point from the segmented cloud is queried within a density-aware assignment radius (Equation 13)

(13) $$r_{\text{assign}}=\alpha d_{\text{med}},$$

where $d_{\text{med}}$ is the median nearest-neighbor spacing among visible splats and α≥1 is a fixed scale. Splats within this radius inherit the semantic and instance labels of the nearest reference point. Unassigned splats are retained for diagnostics.

The mapped splats preserve their original Gaussian parameters (position, spherical harmonic coefficients, opacity, and covariance). Each organ instance is then exported as an individual PLY file containing its associated Gaussian splats, in a format compatible with standard 3DGS renderers. This representation enables per-organ manipulation, rendering, and analysis, while preserving the photometric fidelity of the original reconstruction.

### 2.5 Phenotypic trait quantification

#### 2.5.1 Metric scaling and coordinate frame alignment

With organ-level segmentation complete and all leaf and stem instances mapped back into the 3DGS representation, the resulting labeled plant structures provide the foundation for computing biologically meaningful phenotypic traits. All trait measurements are reported in metric units and within a consistent world coordinate frame. A per-scene scale factor s is obtained from a visible calibration reference; the greenhouse rack width (42 cm) is measured in the SfM reconstruction and compared with its ground-truth dimension to compute the metric scale.

To ensure geometric consistency across scenes, a Manhattan-world alignment is estimated from the rectilinear structural regularities of the greenhouse environment, following the approach of COLMAP’s model orientation aligner (Schönberger and Frahm, 2016; Coughlan and Yuille, 2000). This yields a similarity transform, where R is a rotation matrix aligning the vertical axis with gravity and flattening the ground plane to z = 0. Each Gaussian center is then transformed as. The resulting metric-aligned coordinates form the basis for all downstream trait computations.

#### 2.5.2 Trait definition and computation

We extract six phenotypic traits relevant to muskmelon growth assessment: plant height, leaf surface area (LSA), leaf area index (LAI), leaf count, node count, and internode length. All measurements are computed in the aligned metric frame.

Plant height is defined as the vertical extent from the ground plane (z = 0 in the metric-aligned frame) to the plant apex (Equation 14):

(14) $$H=s\cdot \underset{i}{\text{max}}z_{i}^{\prime }.$$

A small morphological closing operation is applied on the height map to suppress isolated spurious peaks near the apex. The structuring element radius is set to twice the median nearest-neighbor spacing for robustness across scenes.

LSA is computed as the sum of one-sided areas, and LAI normalizes LSA by the fixed reference area $A_{\text{plot}}$. The reference area $A_{\text{plot}}$ used for LAI normalization was fixed at 0.036 $m^{2}$, derived from the mean canopy spread diameter (0.214 m, measured physically with a tape measure) using a circular-footprint approximation. For each leaf instance, we fit a PCA plane at its centroid to define local axes. Each Gaussian ellipsoid belonging to the leaf is orthogonally projected onto this plane, yielding a set of 2D ellipses. The leaf area is computed as the α-shape area of these ellipses, which captures fine leaf geometry while excluding void regions (Equation 15).

(15) $$\text{LSA}=s^{2}\sum_{l=1}^{L}A_{\alpha }(E_{l})\;,\text{ LAI}=\frac{\text{LSA}}{A_{\text{plot}}},$$

Leaf count is the number of leaf instances after simple post-processing that merges leaf fragments whose centroids lie within one median nearest-neighbor spacing and removes components smaller than 1 cm^2^ (in metric scale). This ensures biologically meaningful leaf instances while suppressing artifacts.

A contracted centerline is extracted from the stem subset by Laplacian-based contraction with density-adaptive spacing. An orientation-aware minimum spanning tree (MST) is then constructed over the contracted nodes, and the main axis is selected as the path with maximum vertical span. Nodes are defined at topological junctions along this axis. Internode lengths are computed as geodesic distances between consecutive nodes along the centerline, which provides stable measurements even for curved stems.

## 3 Results and discussion

### 3.1 Optimizing 3DGS scene reconstruction

#### 3.1.1 Efficient frame selection for sTable 3DGS reconstruction

The reconstruction pipeline begins with dense video capture: a 30-second video at 30 frames per second (fps) yields ~900 frames per greenhouse row, covering ~5 meters of crop area. Processing all frames through structure-from-motion (SfM) requires 6–8 hours per scene, making full-resolution reconstruction computationally impractical. To balance reconstruction quality and efficiency, we evaluated three frame-subsampling strategies: Uniform sampling (even temporal spacing), Sharpness-based sampling (ranked by Laplacian variance), and Coverage + Sharpness sampling (enforcing spatial coverage while prioritizing sharp frames).

Reconstruction quality was assessed using two standard SfM geometric metrics from COLMAP: reprojection error (camera pose accuracy) and mean track length (multi-view feature consistency). These metrics directly affect the stability of subsequent 3DGS training, where inaccurate poses or weak correspondences can lead to geometric drift. The comparison of frame-selection strategies for 3DGS reconstruction is detailed in **Table 2**.

**Table 2:** Comparison of frame-selection strategies for 3DGS reconstruction.

Policy		Sharp med. ↑	Sharp σ ↓	TrackLen	Reproj (px)
50%	All Frames	345.22	59.64	8.81	1.266
	Uniform	344.45	58.44	6.40	1.174
	Coverage+Sharpness	364.79	56.59	6.52	1.093
	Sharpness	382.39	33.60	7.74	1.039
25%	Uniform	324.36	57.78	4.99	1.295
	Coverage+Sharpness	389.33	52.79	6.89	1.044
	Sharpness	404.89	28.22	4.21	1.110

As shown in **Table 2**, across six greenhouse scenes. At a 50% sampling rate, the Sharpness strategy achieves the lowest reprojection error (1.039 pixels). At a more aggressive 25% sampling rate, where computational savings are more substantial, the Coverage plus Sharpness maintains comparably low reprojection error (1.044 pixels) while producing substantially longer feature tracks (6.89 versus 4.21), indicating stronger multi-view geometric consistency. This suggests that when subsampling heavily, enforcing spatial coverage is crucial for maintaining reconstruction quality.

Based on these results, we adopt Coverage + Sharpness at 25% sampling for all subsequent reconstructions. This configuration reduces SfM processing time to 1.5-2.0 hours per scene while retaining the geometric stability required for reliable 3DGS optimization. The selected frames serve as input to the plant extraction and phenotyping pipeline described in the following sections.

#### 3.1.2 Reconstruction quality comparison

**Figure 3:**
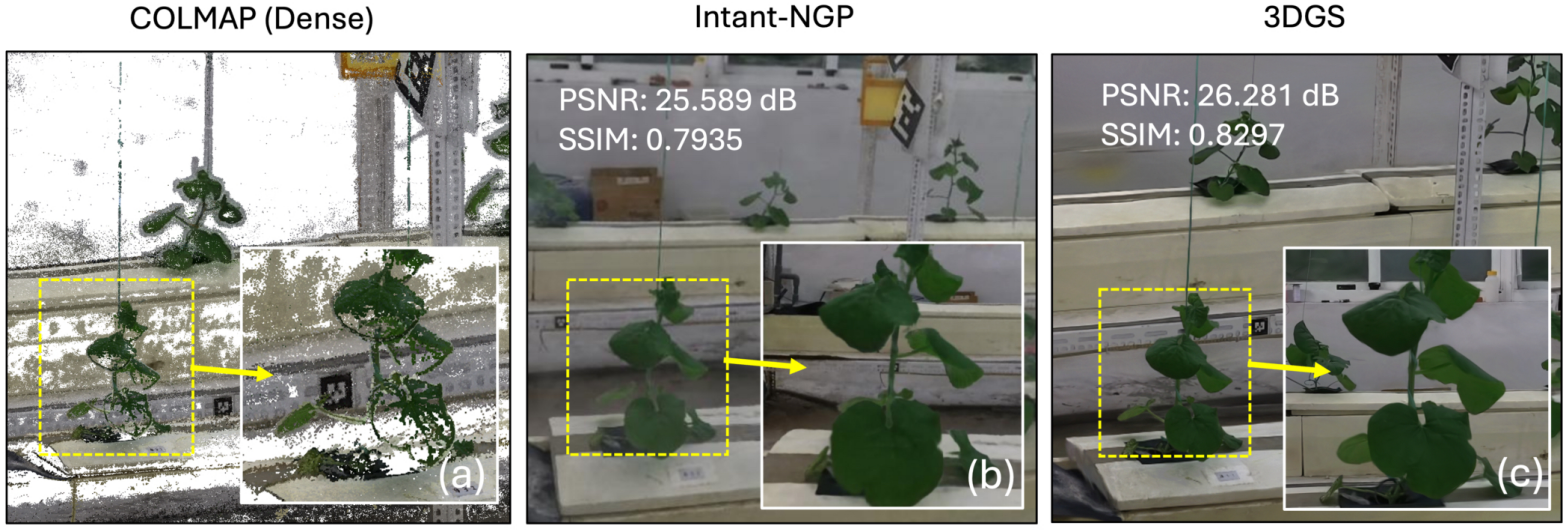
Comparison of reconstruction results from **(a)** dense MVS, **(b)** NeRF, and **(c)** 3DGS. Insets show zoomed regions highlighting differences in geometric and photometric fidelity.

**Figure 3** compares reconstruction results of a representative greenhouse scene using three methods: (a) COLMAP dense reconstruction (shown for qualitative reference), (b) Instant-NGP (NeRF model), and (c) 3D Gaussian Splatting (3DGS). Both neural rendering methods were trained with the same COLMAP-derived camera poses and the selected frame subset determined in Section 3.1.1.

To quantify reconstruction fidelity, Peak Signal-to-Noise Ratio (PSNR) and Structural Similarity Index (SSIM) were computed on a held-out view. Instant-NGP achieves 25.589 dB/0.7935 SSIM, whereas 3DGS attains higher fidelity at 26.281 dB/0.8297 SSIM. In addition to these numerical gains, 3DGS produces visibly sharper foliage and vine structures, preserving leaf shape and thin features that appear blurred or fragmented in the NeRF output. The inset zoom views in **Figure 3** highlight these differences most clearly, showing improved edge integrity along leaf margins and better reconstruction of fine background textures.

Overall, 3DGS provides superior geometric and photometric detail compared with NeRF under identical pose and training conditions, making it a more suitable reconstruction backbone for downstream plant extraction and phenotyping tasks.

### 3.2 Plant instance extraction from 3DGS scenes

#### 3.2.1 Effect of input cue count on plant separation quality

The LCR-GS extraction pipeline allows users to adjust the number of seeded 2D cues per plant, trading annotation effort against separation quality. To quantify this effect, we evaluated three cue configurations on a representative greenhouse scene: one cue (N = 1), three cues (N = 3), and five cues (N = 5). This comparison serves as a single-scene diagnostic to motivate the cue-count setting; formal extraction benchmarking is reported on the full validation set.

**Figure 4:**
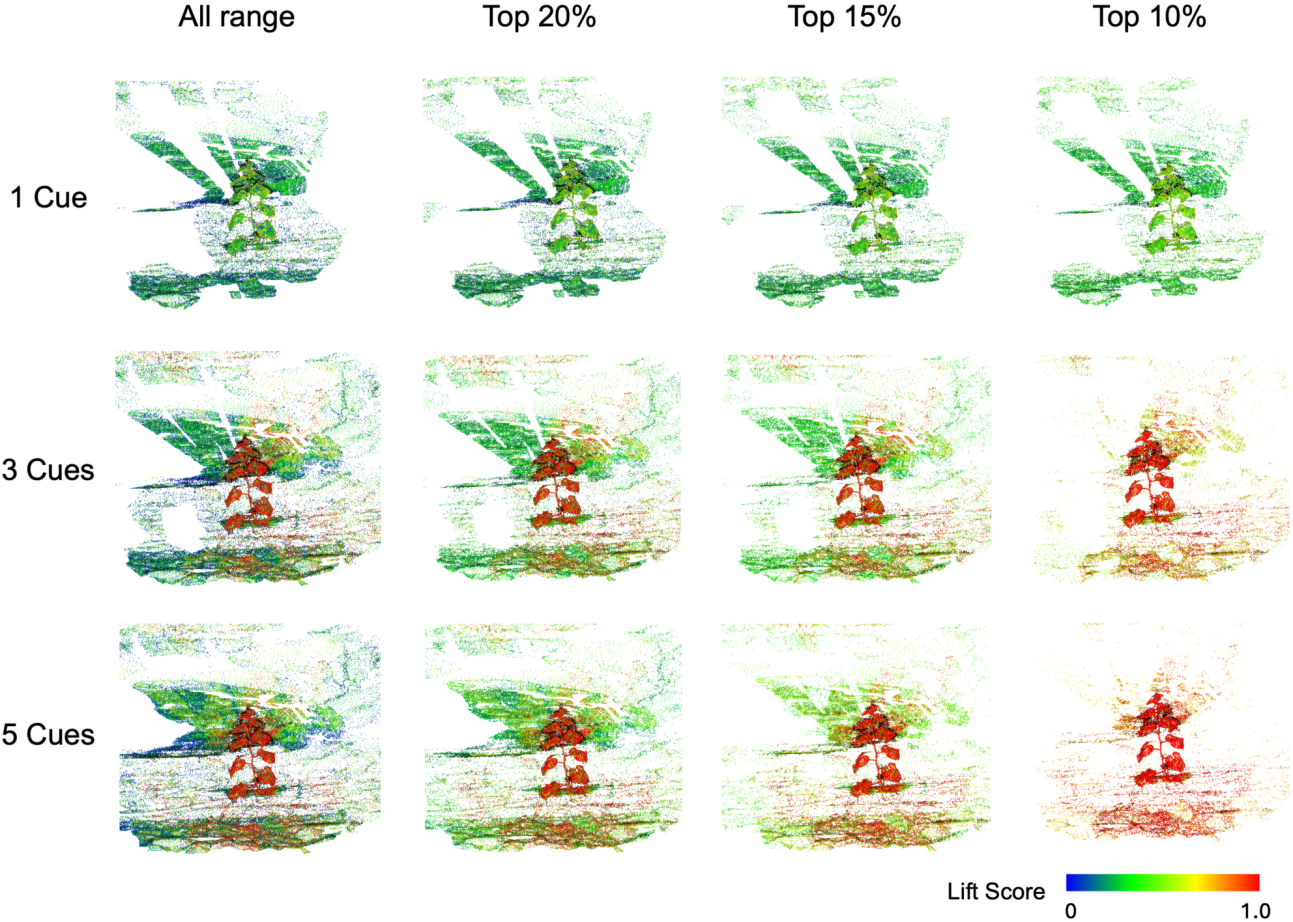
Effect of input cue count (rows) and lift-score retention percentile (columns) on plant-background separation. Higher cue counts and stricter percentiles better isolate high-confidence plant regions.

For each configuration, the per-splat lift scores were independently normalized to the range [0, 1] to allow fair comparison of spatial patterns across different N. **Figure 4** visualizes the spatial distribution of lift scores under the percentile-based thresholds (top 10%, 15%, and 20%). Using percentile thresholds rather than absolute cutoffs enables direct comparison of the separation patterns produced by each cue configuration.

The results reveal distinct separation trends across cue configurations: with one cue (N = 1), plant and background splats exhibit substantial overlap (plants ≈ 0.3-0.8; background ≈ 0.2-0.6), producing ambiguous boundaries and weak clustering performance. Using three cues (N = 3) substantially improves separation, with plant splats consistently scoring above 0.6 and background splats below 0.4, resulting in cleaner boundaries and stronger spatial consistency. Increasing to five cues (N = 5) further expands the high-score regions spatially but reduces peak sharpness, indicating that while additional cues enhance coverage, they yield diminishing returns relative to the increased annotation effort. For all subsequent experiments, we therefore adopt N = 3 cues per plant as a balanced configuration between effort and quality.

#### 3.2.2 Ablation analysis of LCR-GS components

**Figure 5:**
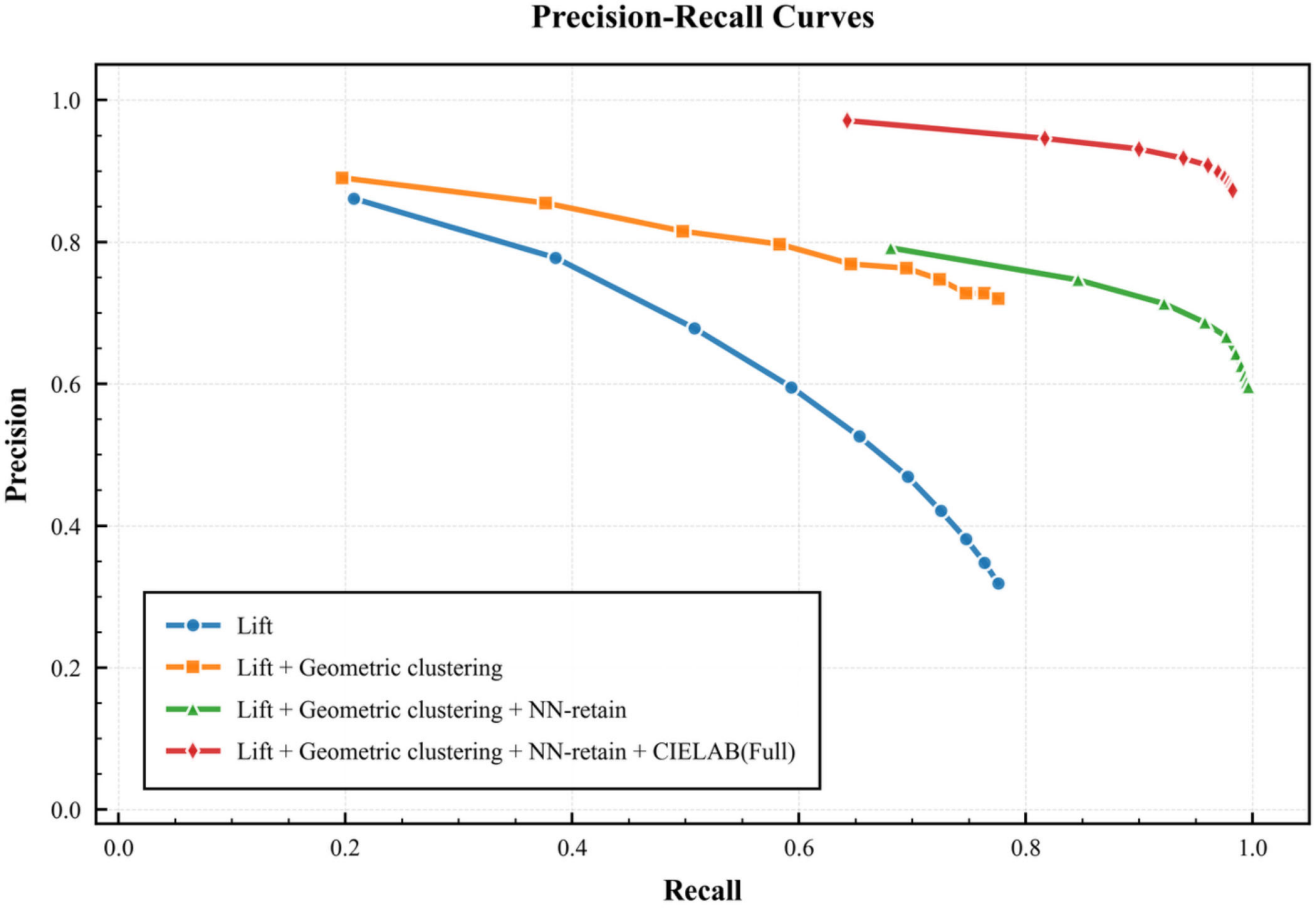
Precision–recall curves showing the incremental effect of each LCR-GS component. Each curve adds one processing stage, illustrating improvements from lifting, geometric clustering, NN-retain, and final CIELAB refinement.

To quantify the contribution of each stage within the LCR-GS pipeline, we conducted a component-wise ablation study on a fixed five-plant diagnostic subset selected across the phenotyping scenes to span plant-size variation, with quantitative results shown in **Figure 5**. Precision-Recall (P-R) curves were generated by varying the lift-score threshold, using percentiles ranging from the top 2% to 20%.

The baseline, represented by the Lift score alone, establishes an initial level of separation (blue curve). While precision is high (0.86) under strict thresholds, it collapses to 0.19 as the threshold relaxes, indicating that lift alone cannot reliably distinguish the target plant from high-scoring background splats. Adding geometric clustering substantially stabilizes precision (orange curve), maintaining a narrower 0.72–0.89 range. This demonstrates that enforcing spatial coherence effectively suppresses distant noise clusters that the baseline approach fails to remove.

Introducing the NN-retain stage improves recall, recovering thin or sparsely represented structures (e.g., thin leaves) that lie near the main cluster (green curve). This process increases the maximum recall to 0.996 while moderately reducing precision (0.596–0.792), reflecting the expected trade-off between completeness and purity.

Finally, applying the CIELAB chromatic refinement yields the best overall performance (purple curve). By leveraging the high-fidelity color representation inherent to 3DGS, this stage removes remaining non-plant splats that are geometrically close but chromatically distinct. The full pipeline achieves a precision range of 0.873–0.971, with only a small reduction in maximum recall. Collectively, these results show that LCR-GS provides a well-balanced extraction strategy with both high precision and high recall.

**Figure 6:**
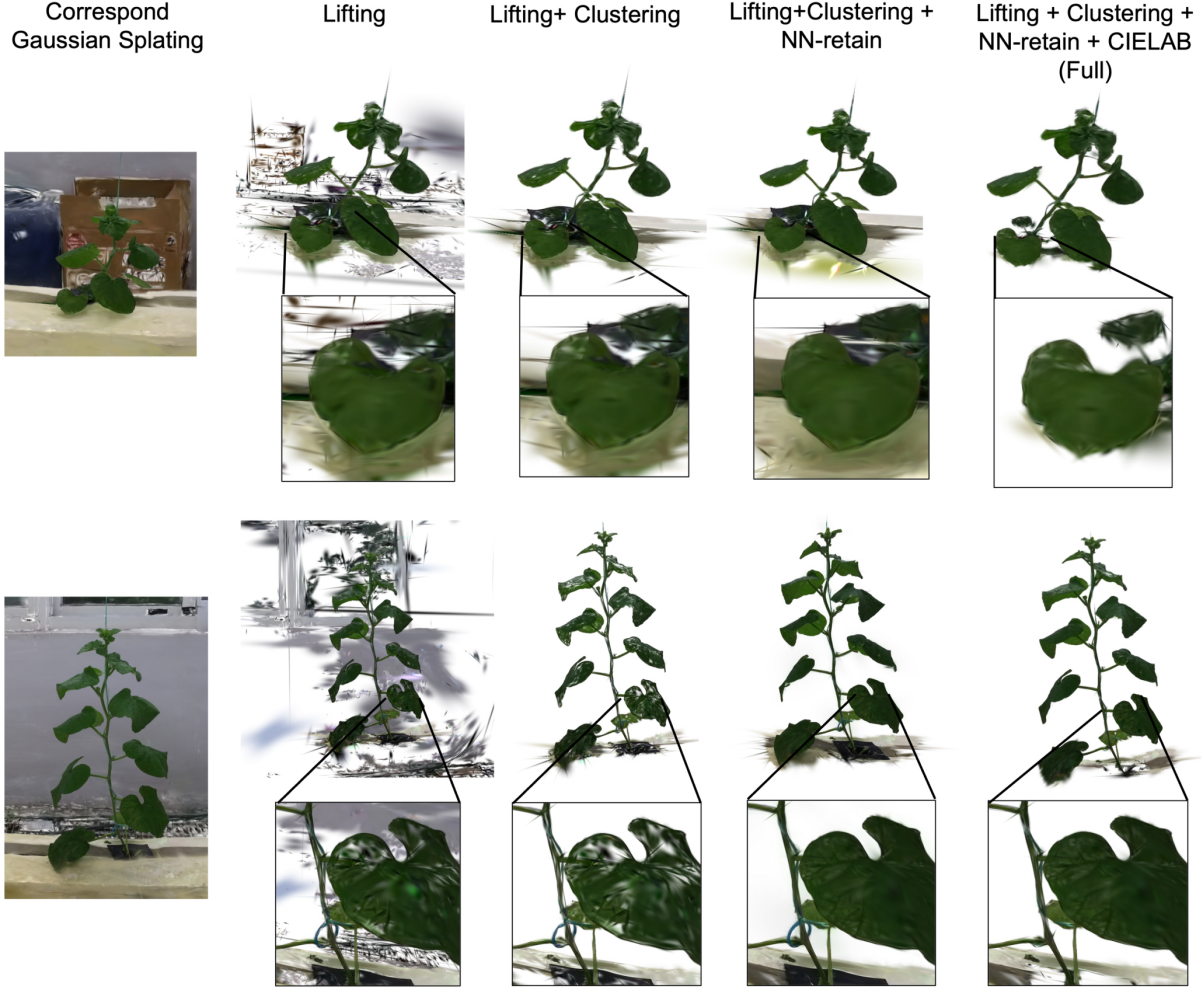
Ablation results on two plants. Columns show the incremental effects of each LCR-GS stage, with insets highlighting improvements in leaf completeness and background removal.

**Figure 6** presents a qualitative visualization of this ablation process at a fixed operating point (top 12% of lift score), selected near the precision inflection point of the full pipeline. These visualizations confirm the quantitative trends: the initial Lifting stage separates the foreground but creates perforations (“holes”) on the leaf surfaces where low-confidence Gaussians were discarded; the Clustering stage effectively removes the non-cued, spatially distinct background; the NN-retain stage then visibly “patches” the leaf perforations, corresponding to the recall gain in **Figure 5**, though it also incorrectly re-includes some non-plant elements near the roots. Before refinement, leakage occurs in two scenarios: (i) loose mask boundaries that include adjacent non-plant regions, and (ii) occluded greenhouse structures (e.g., trellises) in close spatial proximity to the plant. The CIELAB refinement successfully removes these last geometrically-proximate but chromatically-distinct artifacts, yielding a clean and geometrically complete plant instance.

#### 3.2.3 Quantitative and qualitative evaluation of plant-level extraction

To evaluate the performance of the LCR-GS pipeline, we benchmark it against a training-based 2D-lifting approach adapted from the Gaussian Grouping framework (Ye et al., 2024). To ensure a fair comparison, we retrained Gaussian Grouping using the same 2D cues (YOLO-World detections and SAM masks) used by LCR-GS. This isolates the methodological difference between training-integrated grouping (Gaussian Grouping) and post-reconstruction filtering (LCR-GS). The quantitative comparison is summarized in **Table 3**. All methods are evaluated on the full phenotyping validation set (6 scenes, 30 plants) at the fixed top-12% lift-score threshold identified in the ablation study.

**Table 3:** Comparison of plant extraction performance between LCR-GS and a training-based baseline.

Configuration	Precision	Recall	mIoU
Gaussian Grouping	0.381	0.925	0.370
LCR-GS	0.933	0.961	0.890

From **Table 3**, the training-based Gaussian Grouping baseline achieves high recall (0.925) but suffers from very low precision (0.381). This imbalance suggests that the general-purpose segmentation architectures are sensitive to the quality of 2D masks, with the training process incorporating noise and clutter present in the input masks. In contrast, the LCR-GS achieves precision of 0.933 and mIoU of 0.890, demonstrating that its geometric and chromatic filtering stages effectively suppress non-plant Gaussians when the 2D inputs contain clutter or loose boundaries.

In addition to improved accuracy, LCR-GS offers computational advantages. Because it operates after 3DGS reconstruction, it requires no per-scene retraining. The extraction process requires approximately 12 seconds per plant for geometric clustering (OPTICS + NN-retain) and maintains a peak memory footprint of 1.12 GB. The pipeline also performs substantial data reduction: dense greenhouse reconstructions contain an average of 2.1M Gaussians, whereas extracted plant instances contain only 15K-21K Gaussians, a reduction of roughly 99%. This compact representation enables the subsequent organ-level segmentation to run efficiently within standard GPU memory constraints. On an NVIDIA RTX 4080 GPU (16 GB), end-to-end processing for a single greenhouse scene (five plants) requires approximately 2–2.5 h. SfM accounts for most of the runtime; the extraction, segmentation, and trait computation stages together account for the remaining fraction. Structure-from-motion requires approximately 1.5–2.0 h, and 3DGS optimization requires 20–30 min.

**Figure 7:**
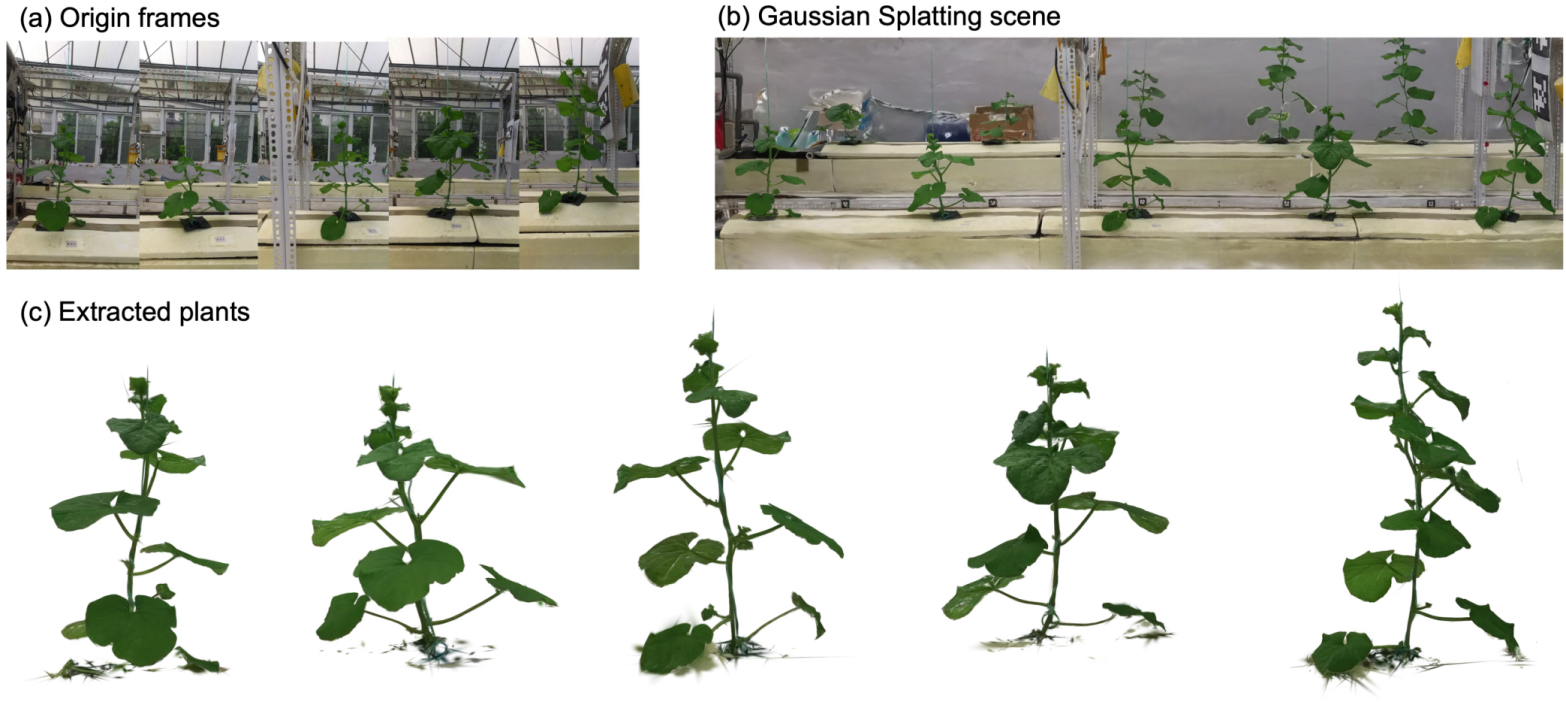
Comparison of **(a)** original RGB frames, **(b)** the reconstructed 3DGS scene, and **(c)** extracted plant instances obtained using LCR-GS.

**Figure 7** illustrates the qualitative performance of the complete pipeline. From the original RGB video frames (**Figure 7a**), the 3DGS reconstruction produces a dense, photorealistic scene (**Figure 7b**). The LCR-GS pipeline isolates individual plant instances (**Figure 7c**), preserving fine leaf structure and stem geometry while cleanly separating the plant from the surrounding greenhouse structures. These results confirm that LCR-GS provides both high-quality plant isolation and compact per-plant representations suitable for downstream phenotypic analysis.

### 3.3 Organ-level instance segmentation performance

To evaluate organ-level segmentation performance, an additional dataset of 140 melon plants was collected using the same acquisition and extraction pipeline described in Section 2. The dataset was split into training (70%), validation (15%), and test (15%) subsets, yielding 98 training plants, 21 validation plants, and 21 test plants. To improve model generalization, training samples were generated using a relaxed CIELAB chromaticity filter (90% quantile), deliberately including a small proportion of non-plant Gaussians. This strategy, which increases plant recall, ensures that fine leaf details are preserved during training. **Table 4** reports performance across three segmentation backbones on the test set, evaluated using Average Precision at IoU 0.5 (AP50) and Average Recall (AR50).

**Table 4:** Organ-level segmentation performance (AP50 and AR50) of different point-cloud instance segmentation models.

Method	AP50			AR50		
	Stem	Leaf	Mean	Stem	Leaf	Mean
PTv3 + PointGroup	0.943	0.905	0.924	0.977	0.927	0.952
PointGroup	0.848	0.903	0.876	0.933	0.927	0.930
SoftGroup	0.836	0.948	0.892	0.867	0.948	0.908

As demonstrated in **Table 4**, the PTv3 backbone achieves the best overall results (mean AP50 = 0.924, AR50 = 0.952), outperforming both PointGroup and SoftGroup. Stem segmentation benefits substantially from PTv3’s long-range attention mechanism, which is essential for modeling the elongated, continuous geometry of vines. Leaf segmentation remains more challenging across all methods due to inter-leaf occlusion and variability in leaf size and curvature.

**Figure 8:**
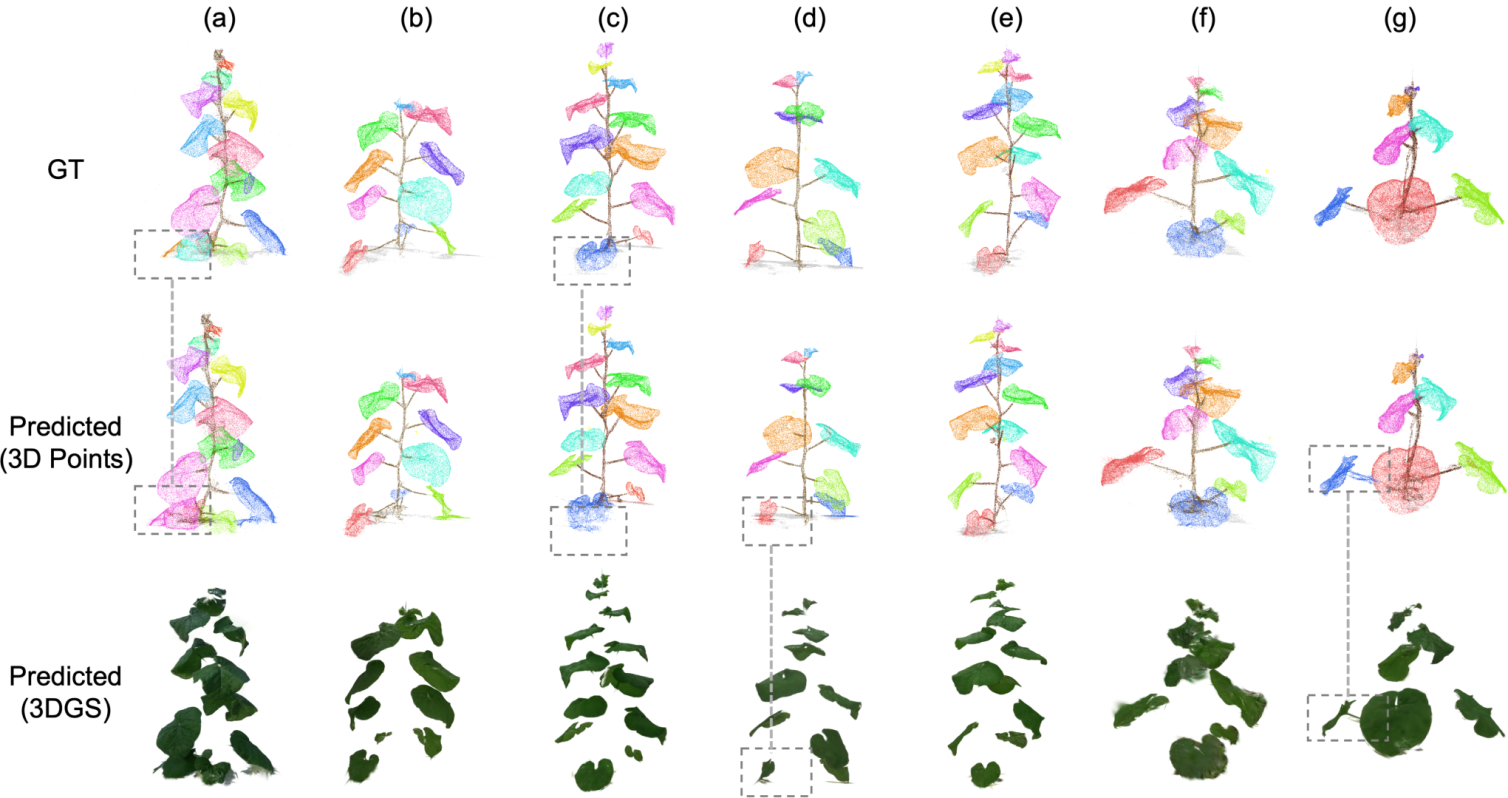
Comparison of organ-level segmentation results across seven representative test plants **(a–g)**. For each plant, the top row shows ground-truth annotations, the middle row shows PTv3 predictions in point-cloud space, and the bottom row shows reconstructed organ instances in the 3DGS representation.

**Figure 8** visualizes segmentation results across seven representative test plants. The top row shows the ground truth annotations with per-instance color coding. The middle row presents inference results from the PTv3 model, demonstrating that most organs are correctly segmented with clear boundaries. A small fraction of leaves exhibits imperfect separation from stems, particularly at attachment points where point density is high and geometric features are ambiguous (plant g). Additionally, in regions where non-plant elements are present, the model must distinguish plant tissue from a structurally similar background based on subtle geometric cues (plant a, c).

The bottom row shows the final results after remapping segmented instances back to the 3DGS representation. Compared to the point cloud segmentation (middle row), the Gaussian-based rendering exhibits cleaner boundaries and reduced noise. This improvement is primarily due to the CIELAB color refinement being reapplied during the remapping stage. This step effectively removes residual non-plant Gaussians by using the strict extraction threshold (80% quantile) defined in the original extraction pipeline.

Furthermore, this remapped 3DGS representation offers unique advantages for post-processing. Properties inherent to the 3DGS format, such as per-Gaussian opacity and explicit ellipsoidal geometry, allow for additional targeted filtering after segmentation. For instance, reconstruction artifacts that manifest as elongated, low-opacity Gaussians (e.g., elongated, low-opacity splats with high axis ratios) could be statistically identified from the final instances, demonstrating a clear advantage of operating in the Gaussian space over point-based methods.

### 3.4 Phenotypic trait validation and population analysis

To evaluate the accuracy of the proposed phenotyping pipeline, trait values computed from the organ-level 3DGS representations were compared against manual measurements. Plant height and leaf count were selected for validation because they are directly measurable and serve as key indicators of early vegetative growth. Manual height was measured from the pot surface to the apical meristem using a ruler, and leaf count was obtained by visually enumerating fully expanded leaves (≥2 cm diameter). All validation results reported in this section are based on the early-vegetative muskmelon cohort (6 scenes, 30 plants).

**Figure 9:**
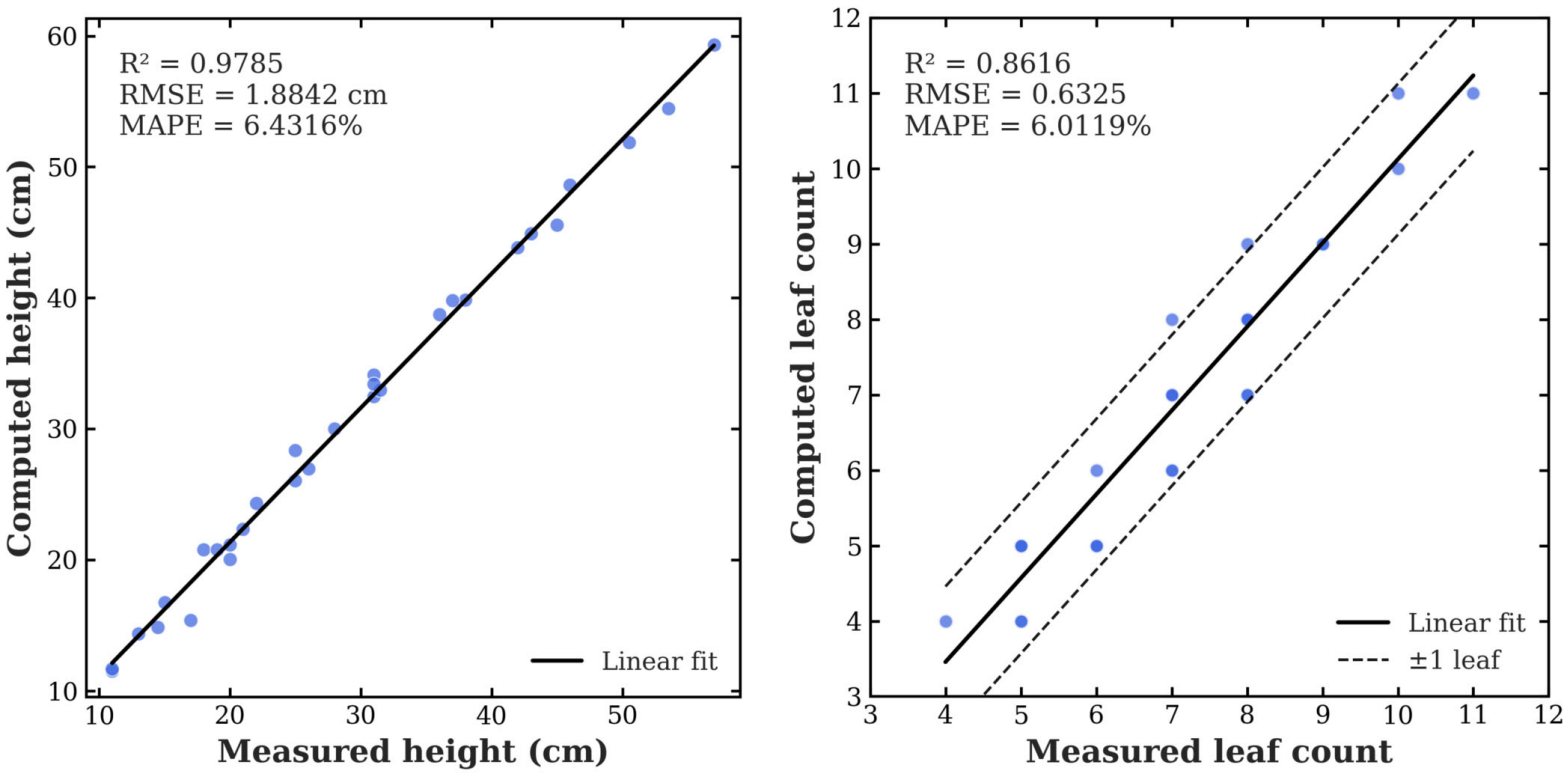
Validation of computed phenotypic traits against manual measurements. Left: correlation between computed and measured plant height. Right: correlation between computed and measured leaf count.

**Figure 9** shows the correlation between computed and measured traits. Plant height exhibits excellent agreement (R² = 0.98, RMSE = 1.88 cm, MAPE = 6.4%), confirming that the 3DGS reconstruction accurately captures vertical plant structure. Statistical analysis reveals a near-constant systematic overestimation (mean residual: +1.58 cm, median: +1.60 cm, p < 10^-8^), with 29 of 30 plants showing positive residuals. Approximately 70% of residuals fall within ±2 cm and 93% within ±3 cm of the ground-truth value. Calibration statistics (slope = 1.025, intercept = 0.852) confirm that this bias is primarily a constant offset rather than a scale distortion. A weak positive association exists between plant size and residual magnitude (Spearman ρ = 0.38, p = 0.039), indicating that taller plants tend to show slightly larger overestimation, though the effect is modest. This offset is attributed to partial inclusion of below-pot stem segments in the reconstructed plant volume. Under the uniform-container conditions of this study, the near-constant offset does not affect inter-plant ranking or relative trait comparisons.

Leaf count shows moderate but acceptable agreement (R² = 0.86, RMSE = 0.63 leaves, MAPE = 6.0%). The mean residual is not statistically significant (−0.20 leaves, t = −1.80, p = 0.083), though calibration analysis reveals a slight negative intercept (−0.986, p = 0.042) and a slope slightly above unity (1.111, p = 0.091), reflecting mild undercounting in sparse canopies and over-counting in dense ones. As shown in **Figure 9** (right), 90% of predictions lie within ±1 leaf, providing sufficient accuracy for population-level phenotyping despite localized segmentation ambiguities (e.g., at leaf–stem junctions or under tight occlusion). Plant height and leaf count are the only traits directly validated against manual measurements in this study. The remaining traits reported in **Table 5** (leaf area, LAI, mean internode length, and stem node count) are pipeline-derived descriptors computed from the segmented 3DGS representations without independent physical verification; they are reported for within-cohort comparison under the assumption of consistent estimation across plants. Population-level trait statistics are summarized in **Table 5**.

**Table 5:** Plant Height and Leaf Count are validated against manual measurements; all other traits are pipeline-derived descriptors reported for within-cohort comparison and not claimed as independently validated measurements.

Trait	Level	Mean ± STD	Range	CV(%)	Unit
Plant Height	Plant	31 .4±13.3	11.5-59.3	42.5	cm
LAI	Plant	1.45±0.25	0.91-2.04	17.1	$m^{2}/m^{2}$
Avg. Leaf Area	Leaf	74.9 ±22.8	39-122	30.5	$cm^{2}$
Leaf Count	Plant	6.9±2.1	4-11	30.8	count
Node Count	Stem	6.9±3.2	3-16	47.0	count
Avg. Internode Length	Stem	3.7±0.9	1.9-6.1	23.6	cm

As depicted in **Table 5**, substantial morphological diversity is observed across the 30-plant cohort: the coefficient of variation ranges from 17.1% (LAI) to 47.0% (stem node count). This variation may reflect genotypic differences, microenvironmental heterogeneity, and stochastic developmental processes. Height and node count show the widest absolute ranges (47.8 cm and 13 nodes), whereas LAI and mean internode length vary more modestly (1.13 m²/m² and 4.2 cm).

Because leaf-area-related traits depend on the geometric suitability of the extracted leaf instances, we performed an internal robustness analysis for area estimation. Of the 224 extracted leaf instances, 204 (91.1%) satisfied geometric screening criteria combining minimum point support (lowest-support 5% excluded), spatial connectivity, and projection planarity; repeating the plant-level area analysis after excluding the remaining 20 instances yielded highly consistent within-cohort rankings. The default α-shape estimator also showed high rank agreement with alternative projected-area methods, and plant-level rankings remained stable across tested α parameter values above the default setting. These analyses support the internal consistency of the area estimator for within-cohort comparison but do not substitute for independent physical validation of the specific workflow used here.

**Figure 10:**
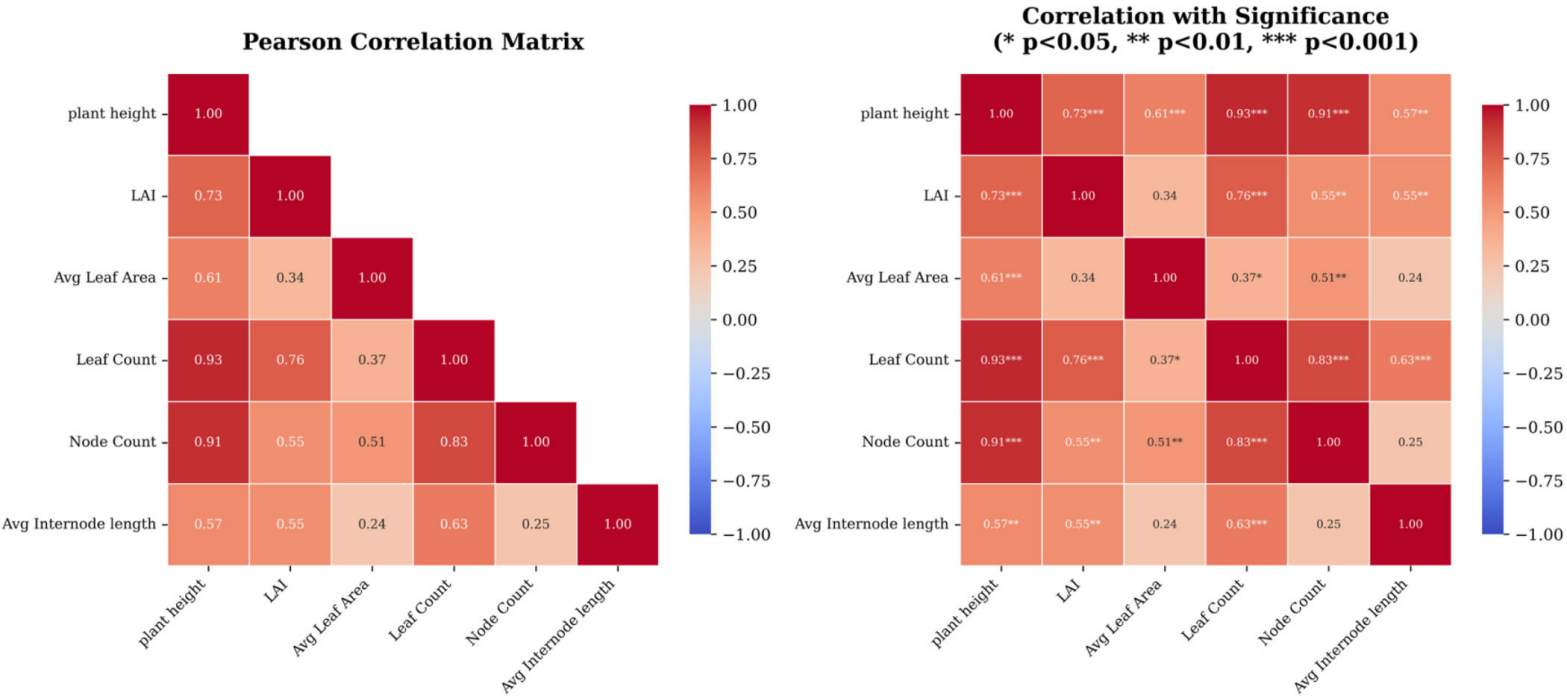
Pearson correlation matrices of organ- and plant-level traits at the vegetative stage, with significance levels indicated on the right panel.

The trait estimates nevertheless permit examination of within-cohort inter-trait relationships (**Figure 10**). Size-related traits display strong correlations - height with leaf count (r = 0.93), node count (r = 0.91), and LAI (r = 0.73) - indicating a coherent growth axis characteristic of early vegetative development. In contrast, average leaf area showed weaker associations with other traits (r = 0.61 with height, r = 0.37 with leaf count), indicating that while overall plant size (height, node count) represents a coordinated growth axis, individual leaf size is a partially independent trait. These trait distributions and correlations provide a basis for downstream phenotypic analyses, including growth modeling, quantitative trait locus mapping, and genotype-by-environment interaction studies.

### 3.5 Scope, limitations, and future directions

**Figure 11:**
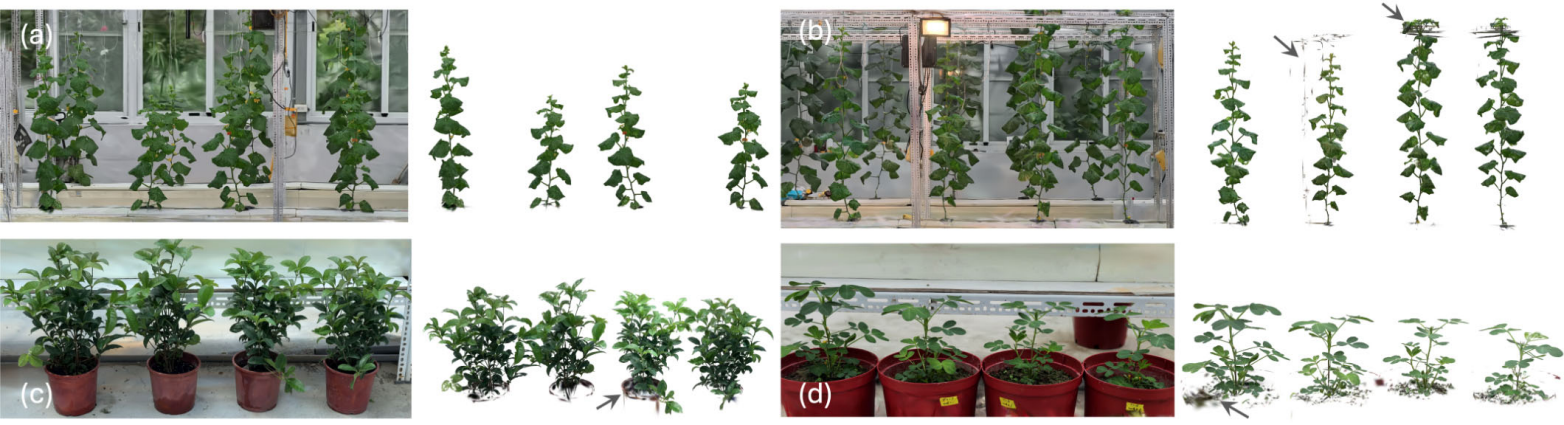
Qualitative examples of LCR-GS extraction beyond the validated setting. Left: representative 3DGS scene renderings; right: corresponding extracted plant instances. **(a)** Mid-stage greenhouse muskmelon. **(b)** Late-stage greenhouse muskmelon, with arrows indicating representative residual or locally incomplete-separation regions. **(c)** Sweet olive and **(d)** peanut, with arrows indicating representative non-target residual regions.

In practical phenotyping systems, scene reconstruction and trait extraction are often treated as separate stages, yet downstream analyses typically assume pre-isolated plant geometry. The proposed LCR-GS framework addresses this gap by introducing an intermediate extraction stage that converts shared-scene 3DGS reconstructions into analysis-ready plant-level units, improving interoperability with downstream organ-level segmentation and trait computation under structured greenhouse acquisition. In this study, quantitative validation in Section 3.4 is limited to early-vegetative greenhouse muskmelon. To examine how the extraction stage behaves beyond that validated regime, we applied LCR-GS to a mid-stage muskmelon scene, a later-stage scene with increased support-structure entanglement, and two additional potted crop settings (**Figure 11**). In the mid-stage muskmelon case, individual plant representations could still be visually distinguished. In the later-stage scene, however, plants extended beyond the trellis height and intertwined with support structures, leading to partially incomplete separation in entangled regions; this marks the current applicability boundary of the present workflow. Preliminary examples in sweet olive (*Osmanthus fragrans*) and peanut (*Arachis hypogaea*) further highlighted that residual noise patterns differed with plant morphology and cultivation setup. Pot-boundary residues, soil-associated points, and the retention or removal of woody stems depended on the intended phenotyping target, identifying task-specific filtering adjustment as one likely challenge in other crop settings.

The validated trait results also require bounded interpretation. Plant height and leaf count remain the only directly validated traits in this study, whereas leaf area and LAI should be read as bounded pipeline-derived descriptors whose values have not been verified against independent physical measurements. Within the uniform-container muskmelon setting studied here, the height residual behaves largely as a near-constant positive offset and is therefore absorbed in relative comparisons. This interpretation is scope-conditional, because changes in container configuration or basal occlusion would make the offset non-constant and require explicit pot-boundary detection or comparable container-aware correction for absolute comparisons. Independent physical validation of leaf-area-related traits remains an important future priority and would require destructive or otherwise independent reference measurements, as in Usenko et al. (2025).

Beyond the structured greenhouse regime studied here, field deployment would require handling uncontrolled illumination, the absence of rectilinear structural cues currently used for scene alignment, and denser plant interactions. Longitudinal use would additionally require reliable temporal registration across time points under canopy change. Broader cross-crop use would likewise require further study of task-specific filtering refinement, as suggested by the distinct residual noise patterns in **Figures 11c, d**.

## 4 Conclusion

This study addressed the challenge of deriving plant-level phenotypic information directly from dense 3D reconstructions of multi-plant greenhouse scenes, a challenge not directly addressed by single-plant acquisition workflows. We introduced an end-to-end pipeline built on 3D Gaussian Splatting (3DGS) and contributed three main advances. First, we established a reconstruction-to-analysis workflow that preserves per-plant geometric fidelity in structured multi-plant greenhouse environments under moderate inter-plant proximity and view-dependent occlusion. Second, we proposed LCR-GS, a post-reconstruction extraction framework that decouples semantics from 3DGS optimization and requires only minimal operator seeding; its Lift-Cluster-Refine stages reliably convert dense scene-level 3DGS models into clean, analysis-ready plant instances. Third, we demonstrated that these extracted plants support robust organ-level segmentation and trait computation, with plant height and leaf count validated against manual measurements and additional morphological traits reported as pipeline-derived descriptors for within-cohort comparison.

Quantitative evaluation on early-vegetative greenhouse-grown muskmelon plants confirmed high extraction precision (0.933), strong segmentation performance (AP50 = 0.924), and close agreement between 3DGS-derived and manually measured plant height (R² = 0.98) and leaf count (90% of estimates within ±1 leaf). These results show that the proposed pipeline achieves accuracy suitable for population-level comparison of the validated traits within the demonstrated cohort while reducing data scale by more than 99% to support efficient downstream learning.

The approach operates within several important constraints. Validation is limited to early-vegetative muskmelon under structured greenhouse conditions; extension to later growth stages, denser canopy architectures, or other crop species requires further study. Leaf area, LAI, and internode-related traits are reported as pipeline-derived descriptors and have not been verified against independent physical measurements. Additional practical limitations include the need for limited manual seeding to initialize multi-view cues and sensitivity to the quality of the underlying 3D reconstruction under challenging lighting or occlusion conditions. Future work will focus on automated cue generation, improved handling of denser canopy conditions, and broader cross-crop applicability with task-specific filtering adjustment.

Despite these constraints, the retained per-plant 3DGS representations open new opportunities for longitudinal monitoring and growth modeling within controlled settings. Because 3DGS encodes explicit geometry and photometric detail, the retained representations provide a useful basis for further downstream analysis. Overall, this work provides a practical path from scene-level 3D reconstruction to organ-level trait characterization for structured greenhouse phenotyping.

## Data Availability

The datasets presented in this study can be found in online repositories. The names of the repository/repositories and accession number(s) can be found below: https://github.com/bblabNTU/3dgs-muskmelon-phenotyping-dataset.
